# Integrated Assessment of HIF-1α and SOD2 Expression and Their Prognostic Implications in Triple-Negative Breast Cancer

**DOI:** 10.3390/ijms27125343

**Published:** 2026-06-13

**Authors:** Burcu Sanal Yılmaz, Sezer Seda Yılmaz, Zeliha Esin Çelik

**Affiliations:** 1 Department of Pathology, Faculty of Medicine, Karamanoğlu Mehmetbey University, Karaman 70200, Turkey; 2 Van Branch of the Council of Forensic Medicine, Van 65080, Turkey; sezersedayilmaz.atk@gmail.com; 3 Department of Pathology, Faculty of Medicine, Selcuk University, Konya 42130, Turkey; dresincelik@hotmail.com

**Keywords:** triple-negative breast cancer, HIF-1α, SOD2, hypoxia, oxidative stress, prognosis

## Abstract

Triple-negative breast cancer (TNBC) is characterized by aggressive clinical behavior and limited targeted therapeutic options. Hypoxia signaling mediated by hypoxia-inducible factor-1α (HIF-1α) and mitochondrial antioxidant defense driven by superoxide dismutase 2 (SOD2) represent biologically interconnected stress-adaptation pathways that may contribute to tumor progression. However, their combined prognostic impact in TNBC remains insufficiently defined. This retrospective study included 70 patients with surgically treated TNBC. Immunohistochemical expression of HIF-1α (nuclear) and SOD2 (cytoplasmic) was semi-quantitatively scored and analyzed individually and in combination. Patients were stratified as both-low, single-high, or both-high expression. Associations with clinicopathological parameters were evaluated, and overall survival (OS) and disease-free survival (DFS) were analyzed using Kaplan–Meier and Cox regression models. Stage-adjusted and stage-free multivariable analyses were performed, and sensitivity analyses using penalized Cox regression were conducted. High SOD2 expression was observed in 68.6% and high HIF-1α expression in 24.3% of cases. Neither marker was independently associated with survival outcomes in Kaplan–Meier or multivariable analyses. HIF-1α-high tumors showed a nominally lower Ki-67 proliferation index compared with HIF-1α-low tumors (median 30.0% vs. 60.0%, *p* = 0.023); however, given the number of comparisons performed, this finding should be regarded as exploratory and interpreted cautiously. Advanced stage and distant metastasis were the strongest predictors of both OS and DFS. In post hoc exploratory analyses, increasing SOD2 (HR 1.36 per point, *p* = 0.039) and HIF-1α scores (HR 1.80 per point, *p* = 0.022) showed nominal associations with worse OS; these findings are hypothesis-generating and should not be interpreted as evidence of independent prognostic value. Combined biomarker stratification showed a numerical stepwise pattern of OS curves, with the poorest survival observed in the both-high group, although statistical significance was not reached. High SOD2 expression is frequent in TNBC and may reflect underlying tumor biology; however, this was not independently confirmed in survival analyses. Continuous biomarker scoring suggests an incremental association with overall survival. Combined HIF-1α/SOD2 assessment did not demonstrate independent prognostic value in this cohort. These observations are hypothesis-generating and require validation in larger, prospectively designed cohorts before any clinical application can be considered.

## 1. Introduction

Breast cancer is a biologically heterogeneous disease, encompassing distinct molecular subtypes with markedly different clinical behaviors and therapeutic vulnerabilities. Among these, triple-negative breast cancer (TNBC) defined by the absence of estrogen receptor, progesterone receptor, and HER2 expression represents one of the most aggressive subtypes, characterized by early recurrence, high metastatic potential, and limited targeted treatment options [1]. Despite advances in systemic therapy, reliable prognostic and predictive biomarkers for TNBC remain insufficient, underscoring the need for a deeper understanding of the biological mechanisms that drive tumor aggressiveness and therapy resistance in this subgroup [2].

One of the defining features of aggressive solid tumors, including TNBC, is the presence of a hypoxic tumor microenvironment. Intratumoral hypoxia arises as a consequence of rapid tumor growth outpacing vascular supply and is strongly associated with poor clinical outcomes, treatment resistance, and metastatic progression [3]. Cellular adaptation to hypoxia is orchestrated primarily by hypoxia-inducible factor-1 (HIF-1), a heterodimeric transcription factor composed of an oxygen-regulated HIF-1α subunit and a constitutively expressed HIF-1β subunit [4]. Under normoxic conditions, HIF-1α is rapidly degraded; however, hypoxia leads to its stabilization, nuclear accumulation, and transcriptional activation of a broad set of genes involved in angiogenesis, metabolic reprogramming, epithelial–mesenchymal transition, invasion, and metastatic niche formation [5].

Accumulating evidence has demonstrated that HIF-1α overexpression in breast cancer is associated with adverse clinicopathological features and unfavorable prognosis, particularly in hormone receptor–negative tumors. Experimental and translational studies have further established HIF-1α as a master regulator of metastatic dissemination, mediating extracellular matrix remodeling and pre-metastatic niche formation through downstream targets such as lysyl oxidase family members. These findings place HIF-1α at the center of hypoxia-driven tumor progression in aggressive breast cancer phenotypes [6,7].

In parallel with hypoxia, oxidative stress represents another critical pressure shaping tumor evolution. Cancer cells generate high levels of reactive oxygen species (ROS) as a byproduct of altered metabolism, oncogenic signaling, and mitochondrial dysfunction [8]. To survive under such conditions, tumors activate antioxidant defense mechanisms, among which superoxide dismutase 2 (SOD2) plays a pivotal role. SOD2 is a mitochondrial enzyme responsible for the conversion of superoxide radicals into hydrogen peroxide, thereby mitigating oxidative damage and contributing to cellular redox homeostasis [9,10,11].

While SOD2 has traditionally been regarded as a tumor suppressor, growing evidence indicates that elevated SOD2 expression in advanced cancers may paradoxically promote tumor progression, therapy resistance, and metastatic competence by enabling cancer cells to adapt to sustained oxidative stress. Recent breast cancer studies have reported that high SOD2 expression is associated with aggressive clinicopathological features, negative hormone receptor status, and poor survival, with particularly consistent overexpression observed in TNBC. Importantly, Kim et al. recently demonstrated in a large TNBC cohort that high SOD2 expression correlates with tumor-infiltrating lymphocyte levels and is associated with prognosis in a stage- and subtype-dependent manner, highlighting the context-dependent and potentially dual role of SOD2 across different tumor microenvironments [11]. However, data regarding the independent prognostic significance of SOD2 specifically within TNBC remain heterogeneous, suggesting that its role is shaped by the broader immunological and hypoxic composition of the tumor microenvironment [9,12,13,14].

Importantly, hypoxia and oxidative stress are not independent phenomena but are biologically intertwined processes. Hypoxia-driven metabolic reprogramming can exacerbate mitochondrial ROS production, while ROS signaling can, in turn, modulate HIF-1α stabilization and activity. Despite this well-established biological crosstalk, the combined impact of HIF-1α–mediated hypoxic adaptation and SOD2-driven oxidative stress tolerance has not been systematically investigated in TNBC. Whether the coexistence of high HIF-1α and high SOD2 expression identifies a distinct, highly adapted and clinically aggressive TNBC phenotype remains largely unexplored [15,16].

Therefore, in the present study, we aimed to evaluate the clinicopathological and prognostic significance of HIF-1α and SOD2 expression—both individually and in combination—in a cohort of patients with triple-negative breast cancer. By integrating hypoxia and oxidative stress markers within the same analytical framework, we sought to better characterize the biological heterogeneity of TNBC and to identify a potential high-risk subgroup defined by simultaneous activation of these adaptive pathways.

## 2. Results

### 2.1. Patient Characteristics and Baseline Clinicopathological Features

A total of 70 patients with triple-negative breast carcinoma (TNBC) were included in the analysis. Within the TNBC cohort, a CK5/6-based immunohistochemical surrogate was used to further classify tumors: 38 patients (54.3%) were classified as CK5/6-positive TNBC, whereas 32 patients (45.7%) were classified as CK5/6-negative TNBC. This dichotomy is used throughout as a practical surrogate subdivision of TNBC and should not be equated with the basal-like transcriptional subtype as defined by gene expression profiling or multi-marker panels (e.g., Nielsen et al.), which additionally require EGFR positivity and broader immunohistochemical criteria. EGFR immunohistochemistry was not uniformly available in this retrospective cohort and could not be incorporated. The baseline clinicopathological and survival characteristics of the cohort are summarized in Table 1.

The median tumor size was 2.6 cm (IQR: 2.0–4.0 cm), and the median Ki-67 proliferation index was 50.0% (IQR: 30.0–70.0). Histological grading demonstrated a predominance of high-grade tumors, with grade 3 accounting for 41 cases (58.6%), followed by grade 2 in 25 cases (35.7%) and grade 1 in 4 cases (5.7%).

Lymph node involvement was present in 32 patients (45.7%), while distant metastasis at diagnosis was identified in 14 patients (20.0%). Lymphovascular invasion (LVI) was observed in 27 cases (38.6%), and perineural invasion was detected in 13 cases (18.6%). Tumor necrosis was frequently present (37 cases, 52.9%), and multifocality was identified in 11 cases (15.7%).

Survival data were available for all patients. The median follow-up duration, estimated using the reverse Kaplan–Meier (Schemper–Smith) method, was 40.5 months (95% CI: 34.2–55.1) for OS and 35.5 months (95% CI: 28.4–48.6) for DFS. During follow-up, 16 patients (22.9%) experienced a DFS event, and 12 patients (17.1%) experienced an OS event (death). The observed median DFS was 35.5 months (IQR: 24.0–60.8) and the observed median OS was 40.5 months (IQR: 26.5–70.8) across the full cohort.

### 2.2. Distribution of SOD2 and HIF-1α Expression

SOD2 immunoexpression was classified as low in 22 patients (31.4%) and high in 48 patients (68.6%). The high prevalence of SOD2-high cases (68.6%) at the ≥3 threshold indicates limited discriminatory capacity of this cutoff in the present cohort, as a large majority of cases are assigned to a single category. HIF-1α immunoexpression was categorized as low in 53 patients (75.7%) and high in 17 patients (24.3%). An important structural feature of the dataset must be disclosed: all 17 HIF-1α-high cases were also classified as SOD2-high, meaning that no HIF-1α-high/SOD2-low cases exist in this cohort. Consequently, the “single-high” group (n = 31) is composed entirely of SOD2-high/HIF-1α-low cases, and the “both-high” group (n = 17) is coextensive with the HIF-1α-high group. The combined stratification therefore does not represent a genuine four-cell 2 × 2 interaction; rather, it reflects a hierarchical stratification in which the SOD2-high subset is further stratified by HIF-1α status. The Spearman correlation between continuous SOD2 and HIF-1α scores was moderate and positive (rho = 0.28, *p* = 0.019), consistent with co-occurrence of high expression but not complete redundancy between the two markers. In combined biomarker stratification, 22 patients (31.4%) were classified as both-low, 31 patients (44.3%) as single-high, and 17 patients (24.3%) as both-high.

### 2.3. Baseline Characteristics According to SOD2 Expression

Clinicopathological characteristics were compared according to SOD2 expression status (Table 2). Tumor size and Ki-67 index were not significantly different between the SOD2-low and SOD2-high groups (tumor size median 3.0 vs. 2.5 cm, *p* = 0.107; Ki-67 median 60.0% vs. 45.0%, *p* = 0.190).

The distribution of CK5/6-positive and CK5/6-negative TNBC, stage group, histological grade, lymph node involvement, and distant metastasis did not differ significantly between SOD2 expression groups (all *p* > 0.05). Similarly, there were no statistically significant differences in the prevalence of LVI, perineural invasion, tumor necrosis, or multifocality between the two groups (all *p* > 0.05).

**Figure 1:**
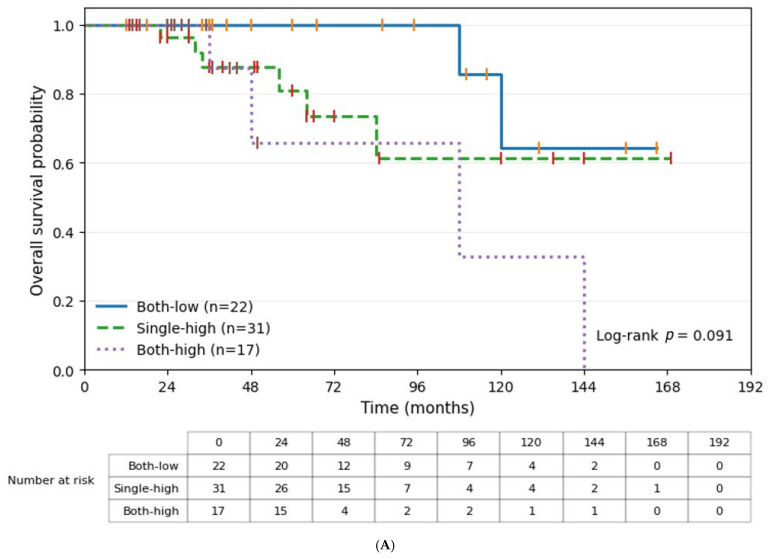

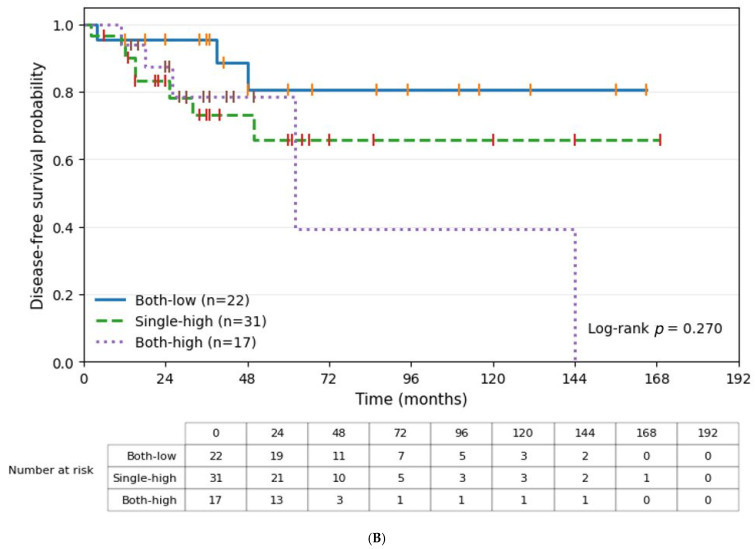
(**A**) Overall survival according to combined HIF-1α/SOD2 expression profile. Kaplan–Meier curves showing overall survival among patients stratified by combined HIF-1α and SOD2 expression status: both-low (n = 22), single-high (either HIF-1α or SOD2 high; n = 31), and both-high (n = 17). Vertical tick marks indicate censored observations. Numbers at risk are displayed below the *x*-axis at 12-month intervals. Survival distributions were compared using the log-rank test (*p* = 0.091). Median follow-up duration was 40.5 months for OS and 35.5 months for DFS, as estimated by the reverse Kaplan–Meier method. (**B**) Disease-free survival according to combined HIF-1α/SOD2 expression profile. Kaplan–Meier curves showing disease-free survival among patients stratified by combined HIF-1α and SOD2 expression status: both-low (n = 22), single-high (either HIF-1α or SOD2 high; n = 31), and both-high (n = 17). Vertical tick marks indicate censored observations (tick marks as orange, purple and red for visual clarity). Numbers at risk are displayed below the *x*-axis at 12-month intervals. Survival distributions were compared using the log-rank test (*p* = 0.270). Median follow-up duration was 40.5 months for OS and 35.5 months for DFS, as estimated by the reverse Kaplan–Meier method.

Survival outcomes by SOD2 expression group are presented through Kaplan–Meier analyses (Figure 1A,B); no statistically significant difference in DFS or OS was observed between SOD2-low and SOD2-high groups by log-rank test (Table 2).

### 2.4. Baseline Characteristics According to HIF-1α Expression

Clinicopathological characteristics were also compared according to HIF-1α expression status (Table 3). Tumor size was numerically smaller in the HIF-1α-high group compared with the HIF-1α-low group; however, this difference did not reach statistical significance (*p* = 0.102).

The Ki-67 proliferation index was lower in the HIF-1α-high group compared with the HIF-1α-low group (median 30.0% vs. 60.0%, *p* = 0.023). However, this finding must be interpreted with caution in light of the multiple-testing burden: approximately 16 comparisons were performed in this table alone, and with an uncorrected α of 0.05, between 0.8 and 1.6 false-positive associations would be expected by chance. No correction for multiple comparisons was applied. To assess robustness, a Spearman correlation between continuous HIF-1α score and continuous Ki-67 values was performed, yielding a rho of −0.21 (*p* = 0.085), which did not reach conventional significance. The Ki-67 association should therefore be regarded as a hypothesis-generating observation rather than a confirmed finding. Other clinicopathological characteristics—including CK5/6-positive and CK5/6-negative TNBC distribution, stage group, histological grade, lymph node involvement, distant metastasis, LVI, perineural invasion, tumor necrosis, and multifocality—were comparable between HIF-1α expression groups (all *p* > 0.05).

Survival outcomes by HIF-1α expression group are presented through Kaplan–Meier analyses; DFS and OS did not differ significantly between HIF-1α-low and HIF-1α-high groups by log-rank test (Table 3).

### 2.5. Association of Clinicopathological Features with Distant Metastasis

Clinicopathological variables were compared according to the presence of distant metastasis at diagnosis (Table 4). Distant metastasis was present in 14 patients (20.0%), while 56 patients (80.0%) had no distant metastasis.

Kaplan–Meier survival analysis demonstrated a marked survival disadvantage in patients with distant metastasis. Overall survival (OS) events occurred in 10 of 14 patients (71.4%) in the metastasis-present group compared with 2 of 56 patients (3.6%) in the metastasis-absent group (log-rank *p* < 0.001). Median OS was not reached in the metastasis-absent group, whereas it was 84.0 months in the metastasis-present group. The estimated 5-year OS was 96.4% in patients without distant metastasis and 35.7% in those with distant metastasis.

Similarly, disease-free survival (DFS) was significantly worse in the metastasis-present group. DFS events occurred in all patients with distant metastasis (14/14, 100%) compared with 2 of 56 patients (3.6%) in the metastasis-absent group (log-rank *p* < 0.001). Median DFS was not reached in the metastasis-absent group but was 18.0 months in the metastasis-present group. The estimated 5-year DFS was 96.4% in the metastasis-absent group and 0% in the metastasis-present group.

As expected, stage distribution differed significantly according to metastasis status. All patients with distant metastasis were classified as advanced stage (III–IV), whereas none of the early-stage patients (I–II) had distant metastasis (*p* < 0.001). Lymph node involvement and lymphovascular invasion were also significantly more frequent in patients with distant metastasis (*p* = 0.006 and *p* = 0.027, respectively). No significant differences were observed regarding histological grade, perineural invasion, tumor necrosis, multifocality, molecular phenotype, or biomarker expression (all *p* > 0.05).

### 2.6. Kaplan–Meier Survival Analyses

To facilitate interpretation of the combined biomarker analyses, patients were stratified into three predefined phenotypic groups according to HIF-1α and SOD2 expression: both-low (low expression of both markers), single-high (high expression of either HIF-1α or SOD2, but not both), and both-high (concurrent high expression of both markers). This classification was designed to reflect increasing activation of hypoxia and oxidative stress adaptation pathways.

Given the limited number of events and the sample size, most comparisons did not reach statistical significance and should be interpreted as exploratory.

Kaplan–Meier survival analyses were performed to evaluate the association of biomarker expression with OS. In the SOD2-based analysis, OS was shorter in numerical terms in the SOD2-high group (log-rank *p* = 0.066). Although shorter overall survival in numerical terms was observed, this difference did not reach statistical significance. Median OS was not reached in either group during follow-up, reflecting the limited number of events (2/22 vs. 10/48). Similarly, median DFS was not reached in both groups, although numerically more events occurred in the SOD2-high group. The estimated 3-year OS was 100% in the SOD2-low group versus 88.1% in the SOD2-high group, and the estimated 5-year OS was 100% versus 77.9%, respectively.

Similarly, no statistically significant difference in OS was observed between the HIF-1α-low and HIF-1α-high groups (log-rank χ^2^ = 3.14, *p* = 0.076). Median OS was not reached in the HIF-1α-low group (8 events/53 patients) but was 108.0 months in the HIF-1α-high group (4 events/17 patients). The estimated 5-year OS was 89.0% in the HIF-1α-low group versus 65.6% in the HIF-1α-high group.

In the combined biomarker analysis, OS demonstrated a numerical stepwise pattern across combined phenotypes, with the most favorable survival observed in the both-low group and the poorest survival in the both-high group (log-rank χ^2^ = 4.80, *p* = 0.091). Median OS was not reached in both-low and single-high groups, whereas it was 108.0 months in the both-high group. The estimated 5-year OS decreased from 100% (both-low) to 80.9% (single-high) and 65.6% (both-high) (Figure 1A).

Kaplan–Meier analyses were also performed for DFS. DFS was shorter in numerical terms in the SOD2-high group; however, this difference did not reach statistical significance (log-rank χ^2^ = 2.45, *p* = 0.118). Median DFS was not reached in either group during follow-up, although numerically more events occurred in the SOD2-high group. The estimated 3-year DFS was 95.5% in the SOD2-low group versus 75.0% in the SOD2-high group. DFS did not significantly differ between HIF-1α expression groups (log-rank χ^2^ = 1.00, *p* = 0.317). Median DFS was not reached in the HIF-1α-low group (11 events/53 patients) but was 62.0 months in the HIF-1α-high group (5 events/17 patients).

In the combined phenotype analysis, DFS curves showed no statistically significant difference (log-rank χ^2^ = 2.62, *p* = 0.270). Median DFS was not reached in the both-low and single-high groups, while it was 62.0 months in the both-high group (Figure 1B).

### 2.7. Cox Proportional Hazards Regression Analyses

#### 2.7.1. Predictors of Overall Survival

Univariate Cox regression analysis was performed to identify predictors of OS. Both biomarkers did not reach statistical significance with OS. High SOD2 expression showed a higher hazard estimate; however, this did not reach statistical significance (HR 3.78, 95% CI 0.82–17.41; *p* = 0.088), HIF-1α-high showed a higher hazard estimate, but this was not statistically significant (HR 2.85, 95% CI 0.84–9.65; *p* = 0.092) (Table 5).

Among clinicopathological variables, distant metastasis was strongly associated with worse OS (HR 38.35, 95% CI 4.87–302.25; *p* = 0.001), and advanced stage (III–IV) was similarly associated with increased mortality risk (HR 9.45, 95% CI 2.05–43.52; *p* = 0.004). Tumor size, Ki-67, grade 3 status, lymph node involvement, and LVI were not significantly associated with OS in univariate models (all *p* > 0.05).

In the multivariable stage-adjusted model (Table 6), advanced stage remained an independent predictor of worse OS (HR 8.78, 95% CI 1.85–41.78; *p* = 0.006), while neither SOD2 nor HIF-1α expression retained statistical significance after adjustment (SOD2-high: *p* = 0.443; HIF-1α-high: *p* = 0.191).

In the stage-free multivariable model (Table 7), neither SOD2-high nor HIF-1α-high expression was significantly associated with overall survival. Although SOD2-high showed an increased hazard estimate, this did not reach statistical significance and should be interpreted with caution.

#### 2.7.2. Predictors of Disease-Free Survival

Univariate Cox regression analysis was performed to identify predictors of DFS. Advanced-stage disease was strongly associated with an increased risk of DFS events (HR 18.76, 95% CI 4.25–82.82; *p* < 0.001). Nodal involvement and LVI showed higher hazard estimates for DFS; however, neither association reached statistical significance (nodal involvement: HR 2.72, 95% CI 0.94–7.84; *p* = 0.064; LVI: HR 2.34, 95% CI 0.85–6.45; *p* = 0.100). Tumor size, Ki-67, and grade 3 status were not significantly associated with DFS (all *p* > 0.05).

High SOD2 expression was associated with a numerically increased risk of DFS events, although this did not reach statistical significance (HR 2.63, 95% CI 0.75–9.29; *p* = 0.133). HIF-1α-high expression was not significantly associated with DFS (HR 1.71, 95% CI 0.59–4.97; *p* = 0.324) (Table 8).

In the multivariable stage-adjusted model (Table 9), advanced stage remained an independent predictor of worse DFS (HR 17.49, 95% CI 3.92–77.96; *p* < 0.001), whereas neither biomarker remained statistically significant after adjustment.

In the stage-free multivariable model (Table 10), distant metastasis emerged as the strongest independent predictor of DFS (HR 264.35, 95% CI 23.65–2954.27; *p* < 0.001), while SOD2 and HIF-1α were not independently associated with DFS.

### 2.8. Early-Stage Subgroup Analysis (Stage I–II, M0)

To reduce the confounding impact of advanced-stage disease and baseline distant metastasis, a prespecified subgroup analysis was conducted among patients with early-stage (AJCC I–II) disease without distant metastasis at diagnosis (n = 47). Event rates in this subgroup were low (OS events = 2; DFS events = 2), limiting statistical power for survival comparisons.

Kaplan–Meier analyses within the early-stage subgroup demonstrated no statistically significant differences in OS according to SOD2 expression (log-rank *p* = 0.191) or HIF-1α expression (log-rank *p* = 0.342). Likewise, the combined phenotype was not significantly associated with OS (log-rank *p* = 0.401).

Similarly, DFS did not differ significantly by SOD2 status (log-rank *p* = 0.185), HIF-1α status (log-rank *p* = 0.351), or combined phenotype (log-rank *p* = 0.397). These findings should be interpreted cautiously given the very low number of events in this subgroup.

In sensitivity analyses using Firth penalized Cox regression, effect directions remained consistent with conventional models, although hazard ratio estimates were attenuated and confidence intervals were narrower, supporting the robustness of the findings.

### 2.9. Continuous Biomarker Score Analysis

In addition to dichotomized (low/high) categorization, SOD2 and HIF-1α immunoexpression were further analyzed as continuous scores (0–9) using Cox proportional hazards regression. This analysis was not prespecified in the original study plan and was performed as a post hoc exploratory analysis to assess whether continuous scoring provides incremental information beyond binary categorization. Results should therefore be interpreted as hypothesis-generating only and are not cited in support of primary conclusions. In univariate analysis for overall survival, higher SOD2 score was nominally associated with increased mortality risk (HR 1.36 per 1-point increase; 95% CI 1.02–1.81; *p* = 0.039). Similarly, higher HIF-1α score showed a nominal association with overall survival (HR 1.80 per 1-point increase; 95% CI 1.09–2.96; *p* = 0.022).

In contrast, continuous SOD2 and HIF-1α scores were not significantly associated with disease-free survival in univariate models (SOD2 score: *p* = 0.389; HIF-1α score: *p* = 0.180).

## 3. Discussion

Triple-negative breast cancer (TNBC) is defined not only by what it lacks—estrogen receptor, progesterone receptor, and HER2 expression—but also by the microenvironmental pressures it creates and exploits. Rapid outgrowth of vascular supply generates intratumoral hypoxia; this hypoxic milieu, in turn, drives mitochondrial metabolic reprogramming that amplifies reactive oxygen species (ROS) production. Cancer cells that survive this dual stress do so by co-activating complementary adaptive programs: hypoxia-inducible factor-1α (HIF-1α) to orchestrate transcriptional responses to low oxygen, and superoxide dismutase 2 (SOD2) to neutralize the resulting mitochondrial superoxide burden. The biological rationale for examining these two pathways together in TNBC is sound; however, the present study did not demonstrate independent prognostic value for either marker individually or in combination. The present study was designed to interrogate this biological framework directly, by assessing HIF-1α and SOD2 expression together—both as individual markers and as a combined biomarker—in a retrospectively assembled cohort of 70 surgically treated TNBC patients, and by examining their joint contribution to clinicopathological risk stratification and survival.

### 3.1. Hypoxia and Oxidative Stress as Interlocked Drivers of TNBC Adaptation

The conceptual foundation of this study rests on the recognition that hypoxia and oxidative stress are not simply co-occurring phenomena in solid tumors, but are co-dependent stress axes that reinforce each other. Under hypoxia, HIF-1α stabilizes and transactivates a broad gene network governing angiogenesis, glycolytic reprogramming, epithelial–mesenchymal transition (EMT), and metastatic niche formation [3,4,5,6,7,17]. This metabolic shift toward anaerobic glycolysis does not eliminate mitochondrial activity; rather, partial electron transport chain function under hypoxia continues to generate superoxide, paradoxically exacerbating intracellular ROS levels. To survive this oxidative pressure, cancer cells upregulate antioxidant defense enzymes, among which SOD2—the primary mitochondrial superoxide scavenger—occupies a central position [9,18,19,20,21]. Crucially, ROS are not passive byproducts: at sub-lethal concentrations, hydrogen peroxide generated downstream of SOD2 can stabilize HIF-1α through prolyl hydroxylase inhibition, creating a positive-feedback loop between hypoxic and redox adaptation programs. This bidirectional crosstalk—hypoxia driving SOD2 upregulation, and SOD2-derived hydrogen peroxide sustaining HIF-1α—means that tumors co-expressing both markers may represent a distinct state of dual microenvironmental adaptation [22,23,24,25,26,27,28]. He et al. demonstrated that acetylated SOD2 accumulating in advanced breast cancer cells stabilizes HIF-2α, promoting cancer stem cell reprogramming and dedifferentiation [9], and Yan et al. showed that HIF-2α directly activates SOD2 transcription through a SOD2–mtROS–endoplasmic reticulum stress axis that confers chemoresistance and stemness properties [26]. Against this mechanistic backdrop, the central contribution of the present study is an integrated, combined analysis of both markers in TNBC, which—to our knowledge—has not been previously reported in a cohort of this design. Recent reviews have further consolidated this mechanistic framework: Bae et al. comprehensively described the interplay between HIF and NRF2 signaling under hypoxia, demonstrating that SOD2-derived ROS serve as a critical second messenger linking oxygen sensing to antioxidant gene transcription [21]. Bigos et al. similarly highlighted that hypoxic tumor microenvironments drive coordinated upregulation of both HIF-target genes and mitochondrial antioxidant programs, with direct implications for therapy resistance in solid tumors including TNBC [22].

### 3.2. SOD2 Overexpression in TNBC: Prevalent, Context-Dependent, and Incrementally Prognostic

High SOD2 expression was observed in 68.6% of our TNBC cohort, a prevalence consistent with the published literature and reflective of the heightened mitochondrial oxidative stress characteristic of this subtype. Kim et al. conducted the most comprehensive immunohistochemical study of SOD2 in TNBC to date, evaluating 229 surgical and 144 biopsy specimens and demonstrating that high SOD2 expression is positively correlated with tumor-infiltrating lymphocyte (TIL) levels and is associated with prognosis in a context-dependent manner [11]. Their findings support a dual role for SOD2 in TNBC: in TIL-high tumors, high SOD2 may paradoxically associate with better immune engagement, while in TIL-low or immunosuppressed contexts, SOD2 overexpression likely reflects a pro-tumorigenic redox adaptation. This immune-stromal interaction provides important biological context for interpreting the null survival findings in our cohort, where TIL data were not available: the prognostic directionality of SOD2 may depend critically on the immunological composition of the tumor microenvironment, a dimension not captured by immunohistochemical scoring alone. Al Haq et al. further showed that SOD2/MnSOD in TNBC functions as a prooxidant peroxidase, increasing mitochondrial ROS and promoting M2 macrophage polarization via the MCT-1/IL-6/NRF2 axis, thereby linking SOD2 to an immunosuppressive and therapeutically resistant TME [24]. It should be noted, however, that the 68.6% high-SOD2 prevalence observed at the ≥3 threshold reflects limited binary discriminatory capacity: when more than two-thirds of cases fall into one category, the cutoff is unlikely to optimally separate biologically distinct subgroups. Sensitivity analyses using the median SOD2 score and tertile-based stratification yielded consistent null findings across all alternative cutoff definitions, suggesting that the absence of a significant survival association is robust to the choice of threshold rather than an artifact of the specific cutoff employed.

In our cohort, SOD2-high tumors did not show a statistically significant difference in disease-free or overall survival by Kaplan–Meier analysis or Cox regression. This null finding is biologically plausible: the dominant prognostic signal in this dataset was disease stage and the presence of distant metastasis, which drove OS events in 71.4% of affected patients. Under such strong confounding, a single molecular marker is unlikely to add independent prognostic weight in a cohort of this size. In a post hoc exploratory analysis, when SOD2 was analyzed as a continuous score, each one-point increase in the staining index was nominally associated with a 36% increase in the hazard of death (HR 1.36 per point, 95% CI 1.02–1.81, *p* = 0.039). This dose–response pattern—which is not captured by high/low cutoffs—is hypothesis-generating and consistent with reports by Papa et al. and Li et al. describing a dose-dependent role for SOD2 in breast cancer progression, particularly in hormone receptor-negative tumors [13,15]. However, as a post hoc, unplanned analysis in a small cohort with limited events, this finding requires independent prospective validation before any interpretive weight can be placed on it. Juliachs et al. further demonstrated that circulating SOD2 levels in breast cancer patients correlate with tumor cell death during neoadjuvant chemotherapy, lending additional biological plausibility to the view that SOD2 expression reflects a functionally active mediator rather than a passive surrogate marker [25].

### 3.3. HIF-1α: Dissociating Hypoxic Aggressiveness from Proliferative Capacity

HIF-1α high expression was detected in 24.3% of tumors—a proportion broadly consistent with prior breast cancer immunohistochemical studies—and was not independently associated with DFS or OS in multivariable models. A nominally lower Ki-67 proliferation index was observed in HIF-1α-high tumors compared with HIF-1α-low tumors (median 30.0% vs. 60.0%, *p* = 0.023). This observation warrants cautious interpretation: given that approximately 16 clinicopathological comparisons were performed in this table without correction for multiple testing, the expected number of false-positive associations by chance alone is 0.8–1.6. The Spearman correlation between continuous HIF-1α score and continuous Ki-67 values was −0.21 (*p* = 0.085), which did not reach statistical significance, further tempering confidence in this association. This finding should therefore be regarded as exploratory and hypothesis-generating only.

If this inverse relationship between HIF-1α and Ki-67 were confirmed in larger cohorts, a plausible biological mechanism could be invoked. Sustained hypoxia promotes cellular quiescence, G1 arrest, and stem-like programming, enabling tumor cells to survive nutrient and oxygen deprivation at the cost of proliferative activity [16,22,23]. In this framework, HIF-1α-high tumors may prioritize invasion, metabolic plasticity, and survival signaling over rapid proliferation. Importantly, Ki-67 is sampled from a single tissue section and reflects local proliferative activity at the time of surgery, not the invasive or metastatic potential encoded in the hypoxic fraction of the tumor. However, until this association is replicated with adequate statistical power and appropriate multiple-testing correction, these mechanistic interpretations remain speculative [4,5,23].

In the same post hoc continuous score analysis, higher HIF-1α scores showed a nominal association with worse overall survival (HR 1.80 per point, 95% CI 1.09–2.96, *p* = 0.022), suggesting that higher-intensity HIF-1α nuclear accumulation—even within the range classified as ‘low’ by dichotomous cutoffs—may carry incremental prognostic information. This pattern parallels the dose–response observation for SOD2 and supports the case for continuous scoring approaches in future biomarker studies. However, as an unplanned post hoc analysis, this finding is exploratory and hypothesis-generating only; it should not be interpreted as evidence of independent prognostic value.

### 3.4. Combined HIF-1α/SOD2 Stratification: An Integrated Biological Framework

The combined biomarker stratification classified patients into three groups—both-low, single-high, and both-high. A critical structural feature of this classification must be acknowledged: all 17 HIF-1α-high cases were also SOD2-high, meaning no HIF-1α-high/SOD2-low cases exist in this cohort. As a result, the “single-high” group consists entirely of SOD2-high/HIF-1α-low cases, and the “both-high” group is coextensive with the HIF-1α-high group. The combined analysis therefore does not represent a genuine two-dimensional interaction between independent markers; rather, it reflects hierarchical stratification within the SOD2-high subset by HIF-1α status. The Spearman correlation between continuous scores was moderate (rho = 0.28, *p* = 0.019), indicating co-occurrence without complete redundancy. With this structural constraint in mind, the observed numerical stepwise pattern in OS curves (5-year OS: 100% → 80.9% → 65.6%; log-rank *p* = 0.091) should be interpreted as largely recapitulating the prognostic gradient associated with HIF-1α expression within the SOD2-high subset, rather than reflecting an independent combined effect.

The biological rationale for co-activation of hypoxic and antioxidant pathways remains scientifically plausible. Yan et al. showed that HIF-2α directly activates SOD2 transcription, reducing mitochondrial ROS and enabling stem-cell reprogramming in drug-resistant breast cancer cells [26]. SOD2-derived hydrogen peroxide can in turn activate NRF2 and amplify HIF stability [21,22,23,24]. These mechanistic links explain why high HIF-1α and high SOD2 co-occur in the same tumors and provide biological motivation for future studies with adequate statistical power to test whether this co-activation carries genuinely independent prognostic information beyond either marker alone.

### 3.5. Stage, Metastasis, and the Prognostic Hierarchy

In formal multivariable models, advanced stage and distant metastasis emerged as the dominant predictors of both OS and DFS, overshadowing the molecular markers after adjustment. This hierarchy is not unexpected and does not undermine the biological relevance of the biomarkers; rather, it reflects the well-recognized challenge that pathological staging absorbs much of the prognostic variance in heterogeneous cohorts that include stage IV disease. In our cohort, all 14 patients with distant metastasis were classified as advanced-stage, and OS events occurred in 71.4% of this group. In the 47-patient early-stage subgroup analysis (stage I–II, M0), event numbers were too low (2 OS events) to draw meaningful conclusions, highlighting that the independent prognostic contribution of HIF-1α and SOD2 may be most apparent in homogeneous, early-stage cohorts with longer follow-up—the appropriate setting for future prospective validation.

### 3.6. CK5/6-Based TNBC Subclassification and the Breadth of Hypoxic/Redox Adaptation

CK5/6-positive TNBC (54.3% of our cohort) has been associated with more aggressive biological features, including enrichment for stemness markers and EMT gene signatures. Despite this, the distribution of HIF-1α and SOD2 expression did not differ significantly between CK5/6-positive and CK5/6-negative TNBC subsets. This finding suggests that hypoxic and redox stress adaptation are not restricted to the CK5/6-positive subset, but represent broader stress-response mechanisms operating across the TNBC spectrum [22]. It must be acknowledged, however, that CK5/6 immunohistochemistry alone is an imperfect and incomplete surrogate for the basal-like transcriptional subtype as defined by gene expression profiling; standard surrogate definitions (e.g., Nielsen et al.) additionally require EGFR positivity, and multi-marker panels are preferred. Because EGFR data were unavailable in this cohort, the CK5/6-positive and CK5/6-negative designations used here should be interpreted as practical subdivisions rather than confirmed molecular subtype assignments. Future studies employing transcriptomic subtyping may more precisely characterize differential engagement of hypoxia and redox programs across TNBC molecular subtypes.

### 3.7. Translational Implications and Future Directions

The biological findings of this study carry potential translational implications that are worth noting strictly in the context of hypothesis generation. HIF-1α inhibitors and hypoxia-activated prodrugs are under active clinical investigation in solid tumors, with TNBC representing a biologically rational target [23]. SOD2 is emerging as a candidate therapeutic vulnerability in TNBC: pharmacological suppression of SOD2 elevates intracellular superoxide to cytotoxic levels and has demonstrated preclinical efficacy [24,29]. Circulating SOD2 has been explored as a non-invasive response biomarker for neoadjuvant chemotherapy [25]. These translational opportunities must be viewed as strictly hypothesis-generating at this stage. The present study does not provide sufficient evidence to support the use of HIF-1α or SOD2 immunohistochemistry for patient selection in clinical trials or routine practice. Any such application requires prospective validation in cohorts with higher event rates, standardized treatment protocols, and comprehensive clinical annotation.

## 4. Materials and Methods

### 4.1. Study Design and Patient Selection

This retrospective cohort study included 70 patients diagnosed with triple-negative breast cancer (TNBC) who underwent surgical resection between 2009 and 2024 at Karamanoğlu Mehmetbey University Research and Training Hospital and Selçuk University Faculty of Medicine. TNBC was defined as <1% expression of estrogen receptor (ER) and progesterone receptor (PR) by immunohistochemistry (IHC), and HER2 negativity (IHC score 0–1+ or IHC 2+ with negative in situ hybridization).

Patients with available formalin-fixed paraffin-embedded (FFPE) tumor tissue and complete clinicopathological data were eligible for inclusion. Cases receiving neoadjuvant chemotherapy prior to tissue sampling, those with insufficient tumor material, extensive necrosis precluding reliable immunohistochemical evaluation, or missing follow-up data were excluded.

Clinicopathological parameters recorded included patient age at diagnosis, tumor size, histological grade, pathological stage (AJCC), lymph node status, presence of distant metastasis, and follow-up outcomes.

### 4.2. Follow-Up and Clinical Endpoints

Patients were followed from the date of initial diagnosis to the date of last follow-up or death. Overall survival (OS) was defined as the time from diagnosis to death from any cause. When available, disease-free survival (DFS) was defined as the time from diagnosis to the first documented recurrence (local, regional, or distant) or death. Patients without events were censored at the last follow-up.

### 4.3. Immunohistochemistry

#### 4.3.1. Tissue Preparation and Staining

Immunohistochemical staining was performed on 4 µm thick sections obtained from formalin-fixed, paraffin-embedded (FFPE) tumor blocks using an automated staining platform (Ventana BenchMark system, Roche Diagnostics, Rotkreuz, Switzerland), ensuring standardized staining conditions across all samples.

Sections were deparaffinized in xylene and rehydrated through graded alcohols. Antigen retrieval was carried out using heat-induced epitope retrieval, with citrate buffer (pH 6.0) for SOD2 and EDTA buffer (pH 9.0) for HIF-1α, according to antibody-specific recommendations.

Endogenous peroxidase activity was blocked using 3% hydrogen peroxide for 10 min at room temperature. Sections were incubated with primary antibodies against SOD2 (rabbit polyclonal antibody, orb499679, Biorbyt, UK) and HIF-1α (mouse monoclonal antibody, clone 28b, sc-13515, Santa Cruz Biotechnology, Santa Cruz, CA, USA). Antibodies were applied at optimized dilutions (SOD2: 1:100; HIF-1α: 1:100) and incubated for 60 min at room temperature.

Following primary antibody incubation, sections were treated with an appropriate secondary antibody and visualized using a diaminobenzidine (DAB) chromogen detection system. Slides were counterstained with hematoxylin.

Positive control tissues included liver and kidney for SOD2 and urinary bladder tissue for HIF-1α, based on manufacturer recommendations. Negative controls were prepared by omitting the primary antibody under identical staining conditions.

#### 4.3.2. Evaluation of Immunostaining

All slides were independently evaluated by two pathologists blinded to clinicopathological and survival data, and discrepant cases were reviewed jointly to reach consensus. Interobserver agreement for the ordinal raw immunoreactivity score (IRS, 0–9) was assessed using linearly weighted Cohen’s kappa, yielding a weighted κ = 0.79 (95% CI: 0.71–0.87) for HIF-1α and weighted κ = 0.81 (95% CI: 0.74–0.88) for SOD2, both indicating excellent agreement. For the final dichotomized classification (high vs. low), standard Cohen’s kappa was κ = 0.82, also reflecting excellent interobserver concordance.

HIF-1α expression was assessed exclusively on the basis of nuclear staining, consistent with its function as a hypoxia-responsive transcription factor, whereas SOD2 expression was evaluated according to cytoplasmic and mitochondrial staining, reflecting its localization as a mitochondrial antioxidant enzyme. For both markers, a semi-quantitative score was calculated by multiplying staining intensity (0 = none, 1 = weak, 2 = moderate, 3 = strong) by the proportion of positive tumor cells, yielding a final score ranging from 0 to 9. The proportion categories were adapted to the biological staining characteristics of each marker.

For HIF-1α, positive tumor cell proportion was scored as 0 (<1%), 1 (1–10%), 2 (11–50%), and 3 (>50%), reflecting the typically focal and heterogeneous nuclear staining pattern observed in hypoxic tumor regions.

For SOD2, positive tumor cell proportion was scored as 0 (<10%), 1 (10–30%), 2 (31–60%), and 3 (>60%), consistent with the generally broader and more diffuse cytoplasmic staining pattern associated with mitochondrial expression.

**Figure 2:**
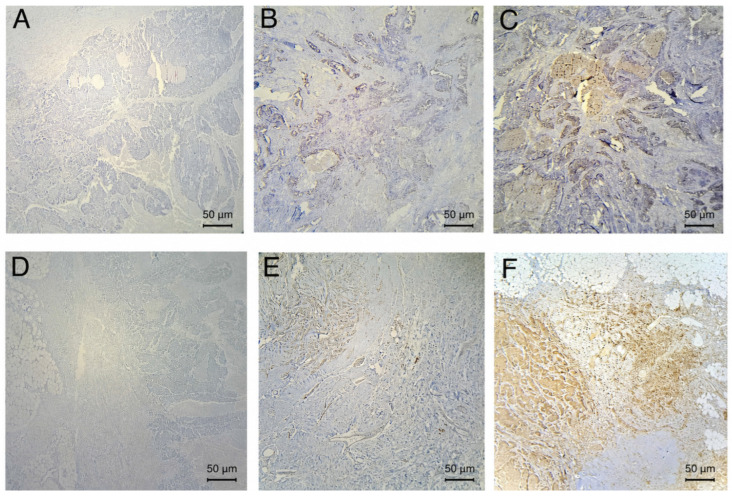
Representative immunohistochemical expression of HIF-1α and SOD2 in triple-negative breast cancer tissues. (**A**) Negative control for HIF-1α showing no specific nuclear staining (×100). (**B**) Low HIF-1α expression with weak and focal heterogeneous nuclear immunoreactivity (×100). (**C**) High HIF-1α expression with strong heterogeneous nuclear staining in a large proportion of tumor cells (×100). (**D**) Negative control for SOD2 showing absence of specific cytoplasmic immunoreactivity (×100). (**E**) Low SOD2 expression demonstrating weak heterogeneous cytoplasmic staining in scattered tumor cells (×100). (**F**) High SOD2 expression demonstrating intense heterogeneous cytoplasmic staining in the majority of neoplastic cells (×100).

HIF-1α was classified as high when the staining index was ≥3, based on previously published breast cancer immunohistochemistry studies and adopted as a prespecified threshold prior to data analysis. To maintain methodological consistency and facilitate integrated analysis, the same cutoff (≥3) was applied to define high SOD2 expression; however, this threshold was not independently validated for SOD2 in the literature and should be regarded as an assumption rather than an evidence-based cutoff for this marker. The implications of this assumption are discussed in the Section 5. Representative immunohistochemical staining patterns, including low and high expression examples and negative controls, are presented in Figure 2.

### 4.4. Combined Expression Analysis

To explore the biological interaction between hypoxia adaptation and oxidative stress response, patients were additionally classified according to combined HIF-1α and SOD2 expression status into three groups:

1. Both markers low;
2. Either HIF-1α or SOD2 high;
3. Both markers high.

This combined analysis aimed to explore whether simultaneous activation of hypoxic and antioxidant pathways identifies a higher-risk TNBC subgroup. It should be noted that in this dataset, all HIF-1α-high cases were also SOD2-high; therefore, the classification is hierarchical rather than a genuine four-cell 2 × 2 combination, and results should be interpreted accordingly.

### 4.5. Ethical Considerations

This study was conducted in accordance with the principles of the Declaration of Helsinki. Ethical approval was obtained from the Ethics Committee of the Faculty of Medicine, Karamanoğlu Mehmetbey University. The study protocol entitled “The Relationship of Superoxide Dismutase-2 (SOD2) and Hypoxia-Inducible Factor-1 Alpha (HIF-1α) Expression with Clinicopathological Prognostic Parameters and Survival in Triple-Negative Breast Carcinomas” was reviewed and approved at the Ethics Committee of Karamanoğlu Mehmetbey University Faculty of Medicine meeting held on 23 September 2024 (Decision No.: 10-2024/11).

Given the retrospective design of the study and the use of archived formalin-fixed, paraffin-embedded tissue samples, the requirement for informed consent was waived by the ethics committee. All clinical data were anonymized prior to analysis, and patient confidentiality was strictly maintained throughout the study.

### 4.6. Statistical Analysis

All statistical analyses were performed using IBM SPSS Statistics v.26.0 (IBM Corp., Armonk, NY, USA) and R software (v.4.3.1; R Foundation for Statistical Computing, Vienna, Austria). Tumor stage was assessed exclusively using the STAGE variable, according to the AJCC staging system, and categorized as early stage (I–II) or advanced stage (III–IV) for analytical purposes. Continuous variables were expressed as median (interquartile range), and categorical variables as frequencies and percentages.

Associations between SOD2 and HIF-1α expression and clinicopathological parameters, including stage group, were evaluated using the χ^2^ test or Fisher’s exact test for categorical variables and the Mann–Whitney U test for continuous variables. Correlations between continuous SOD2 and HIF-1α immunohistochemical scores were assessed using Spearman’s rank correlation coefficient to quantify the degree of co-expression between the two markers; additionally, the Spearman correlation between continuous HIF-1α score and continuous Ki-67 values was computed as a sensitivity analysis to assess the robustness of the categorically observed Ki-67 association. No correction for multiple comparisons was applied to the clinicopathological association analyses; all *p*-values from these comparisons should therefore be interpreted in the context of the number of tests performed (approximately 16 per table), and associations not replicated in independent cohorts should be regarded as exploratory.

Overall survival was analyzed using the Kaplan–Meier method and compared with the log-rank test. Variables significant in univariate analyses were entered into multivariable Cox proportional hazards regression models, with stage group (early vs. advanced) included as a mandatory covariate. Hazard ratios (HRs) and 95% confidence intervals (CIs) were reported.

To address potential model instability due to low event numbers and separation observed in conventional Cox models, sensitivity analyses were performed using Firth’s penalized maximum likelihood estimation for Cox regression, implemented via the coxphf package (version 1.13) in R (v4.3.1). To evaluate the robustness of the primary SOD2 dichotomization, additional sensitivity analyses were conducted using two alternative cutoff definitions: (1) the median SOD2 score as a data-derived cutoff, and (2) tertile-based stratification (low, intermediate, high). Kaplan–Meier and log-rank analyses were repeated under each alternative cutoff definition.

Hazard ratios for tumor size and Ki-67 represent the effect associated with each 1 cm and 1% increase, respectively. Continuous score analyses of SOD2 and HIF-1α (0–9 scale) were post hoc and exploratory; they were not prespecified in the original study plan and should be interpreted as hypothesis-generating only.

Median follow-up duration was estimated using the reverse Kaplan–Meier method (Schemper–Smith estimator), in which censored observations are treated as events and events as censored, providing an unbiased estimate of follow-up time that is not influenced by event occurrence.

A two-sided *p* value < 0.05 was considered statistically significant; all statistical tests performed in this study were two-sided, including χ^2^ tests, Fisher’s exact tests, Mann–Whitney U tests, log-rank tests, and Cox regression analyses.

## 5. Limitations

Several limitations of this study should be acknowledged. First, and most critically, the study spanned a 15-year recruitment period (2009–2024), during which the systemic treatment landscape for TNBC changed substantially—encompassing the introduction of dose-dense anthracycline–taxane regimens, platinum agents, capecitabine in the post-neoadjuvant setting, immune checkpoint inhibitors (pembrolizumab), PARP inhibitors, and antibody–drug conjugates such as sacituzumab govitecan. Because detailed treatment data were not uniformly available in this retrospective cohort, it was not possible to account for therapeutic heterogeneity in survival analyses. This represents a major potential confounder: patients diagnosed in 2009 and patients diagnosed in 2022 may have received markedly different systemic therapies regardless of tumor biomarker status, rendering survival comparisons across the full cohort period difficult to interpret. All survival findings in this study should therefore be interpreted with this limitation explicitly in mind.

Second, the sample size was modest (n = 70), and the number of outcome events was relatively low (12 deaths and 16 disease-free survival events), resulting in limited statistical power and wide confidence intervals in several Cox regression models. Consequently, some associations that demonstrated biologically meaningful effect sizes may not have reached conventional statistical significance.

Third, the retrospective design introduces inherent selection bias and limits control over potentially important confounding factors, including treatment-related variables.

Fourth, HIF-1α immunohistochemistry is particularly susceptible to pre-analytical factors, including tissue fixation time and ischemic delay, and its expression may be markedly heterogeneous within tumors, which may have influenced staining intensity and classification. 

Fifth, although two experienced pathologists independently evaluated all slides and interobserver agreement was high (κ = 0.82), semi-quantitative immunohistochemical scoring remains inherently observer dependent.

Sixth, the CK5/6-positive and CK5/6-negative TNBC classification used in this study is an incomplete surrogate for the basal-like transcriptional subtype. Standard surrogate definitions require CK5/6 and/or EGFR positivity; because EGFR immunohistochemistry was not uniformly available in this retrospective archive, the CK5/6-based dichotomy employed here may misclassify a proportion of cases and does not fully capture the molecular complexity of TNBC subtyping.

Seventh, treatment response variables, including neoadjuvant response and adjuvant therapy details, were not comprehensively available and could not be incorporated into the prognostic models.

Eighth, the ≥3 dichotomization threshold for SOD2 was not independently validated in the literature for this marker and was adopted by analogy from the HIF-1α cutoff for consistency. The resulting classification of 68.6% of cases as SOD2-high reflects limited discriminatory capacity at this threshold. Although sensitivity analyses using the median score and tertile-based stratification yielded consistent null findings, the optimal cutoff for SOD2 in TNBC remains to be established in future studies with larger cohorts and prospective design.

Finally, the study was conducted at two institutions without an independent external validation cohort, limiting the generalizability of the findings. Accordingly, the present results should be considered hypothesis-generating and require confirmation in larger, prospectively designed, multicenter studies with standardized immunohistochemical protocols and comprehensive clinical annotation.

## 6. Conclusions

In conclusion, high SOD2 expression was frequent in TNBC and may reflect underlying tumor biology; however, it was not independently associated with DFS or OS in Kaplan–Meier or multivariable survival analyses. Continuous score analysis demonstrated a nominally significant incremental association with overall survival, but this was a post hoc exploratory finding that requires prospective validation before any prognostic application. HIF-1α expression identified a distinct subset characterized by lower proliferative activity, suggesting that hypoxia-driven aggressiveness may not be fully captured by proliferation markers alone; however, this association did not withstand correction for multiple comparisons and should be interpreted cautiously. Combined HIF-1α/SOD2 assessment did not demonstrate independent prognostic value in multivariable analyses in this cohort. Taken together, these findings are hypothesis-generating and do not support the clinical use of these markers for risk stratification outside of a research context. Confirmation in larger, independent, prospectively designed cohorts with higher event rates and comprehensive treatment annotation is required before any conclusions can be drawn.

## Figures and Tables

**Table 1:** Baseline Clinicopathological and Survival Characteristics of the Cohort (n = 70).

Variable	Category	Overall (n = 70)
Molecular phenotype	CK5/6-positive TNBC	38 (54.3%)
	CK5/6-negative TNBC	32 (45.7%)
AJCC stage	IA	12 (17.1%)
	IB	0 (0.0%)
	IIA	20 (28.6%)
	IIB	15 (21.4%)
	IIIA	4 (5.7%)
	IIIB	4 (5.7%)
	IIIC	1 (1.4%)
	IV	14 (20.0%)
Stage group	Early (Stages I–II)	47 (67.1%)
	Advanced (Stages III–IV)	23 (32.9%)
Tumor size (cm)	—	2.6 (2.0–4.0)
Histological grade	1	4 (5.7%)
	2	25 (35.7%)
	3	41 (58.6%)
Lymph node involvement	No	38 (54.3%)
	Yes	32 (45.7%)
Distant metastasis	No	56 (80.0%)
	Yes	14 (20.0%)
Lymphovascular invasion	Absent	43 (61.4%)
	Present	27 (38.6%)
Perineural invasion	Absent	57 (81.4%)
	Present	13 (18.6%)
Tumor necrosis	Absent	33 (47.1%)
	Present	37 (52.9%)
Multifocality	Absent	59 (84.3%)
	Present	11 (15.7%)
Ki-67 (%)	—	50.0 (30.0–70.0)
Disease-free survival, observed follow-up time (months)	—	35.5 (24.0–60.8)
DFS event	No event	54 (77.1%)
	Event	16 (22.9%)
Overall survival, observed follow-up time (months)	—	40.5 (26.5–70.8)
OS event	Alive	58 (82.9%)
	Death	12 (17.1%)

Data are presented as n (%) for categorical variables and median (interquartile range) for continuous variables. AJCC: American Joint Committee on Cancer; DFS: disease-free survival; OS: overall survival.

**Table 2:** Baseline characteristics according to SOD2 expression.

Variable	Category	Low (n = 22)	High (n = 48)	*p*-Value
Tumor size, cm	Median (IQR)	3.0 (2.5–4.4)	2.5 (1.9–3.6)	0.107
Ki-67, %	Median (IQR)	60.0 (35.0–70.0)	45.0 (28.8–70.0)	0.190
Molecular phenotype	CK5/6-positive TNBC	15 (68.2%)	23 (47.9%)	0.114
	CK5/6-negative TNBC	7 (31.8%)	25 (52.1%)	
Stage group	Early (I–II)	17 (77.3%)	30 (62.5%)	0.221
	Advanced (III–IV)	5 (22.7%)	18 (37.5%)	
Histological grade	1	0 (0.0%)	4 (8.3%)	0.290
	2	7 (31.8%)	18 (37.5%)	
	3	15 (68.2%)	26 (54.2%)	
Lymph node involvement	No	12 (54.5%)	26 (54.2%)	0.976
	Yes	10 (45.5%)	22 (45.8%)	
Distant metastasis	No	19 (86.4%)	37 (77.1%)	0.523
	Yes	3 (13.6%)	11 (22.9%)	
Lymphovascular invasion	Absent	13 (59.1%)	30 (62.5%)	0.785
	Present	9 (40.9%)	18 (37.5%)	
Perineural invasion	Absent	17 (77.3%)	40 (83.3%)	0.529
	Present	5 (22.7%)	8 (16.7%)	
Tumor necrosis	Absent	7 (31.8%)	26 (54.2%)	0.082
	Present	15 (68.2%)	22 (45.8%)	
Multifocality	Absent	18 (81.8%)	41 (85.4%)	0.731
	Present	4 (18.2%)	7 (14.6%)	

Continuous variables are presented as median (interquartile range, IQR). Categorical variables are presented as number (percentage). *p*-values are shown once per variable and correspond to comparisons between the LOW and HIGH SOD2 expression groups.

**Table 3:** Baseline characteristics according to HIF-1α expression.

Variable	Category	LOW (n = 53)	HIGH (n = 17)	*p*-Value
Tumor size, cm	Median (IQR)	3.0 (2.0–4.0)	2.2 (1.7–3.0)	0.102
Ki-67, %	Median (IQR)	60.0 (30.0–70.0)	30.0 (10.0–50.0)	0.023
Molecular phenotype	CK5/6-positive TNBC	30 (56.6%)	8 (47.1%)	0.491
	CK5/6-negative TNBC	23 (43.4%)	9 (52.9%)	
Stage group	Early (I–II)	36 (67.9%)	11 (64.7%)	0.805
	Advanced (III–IV)	17 (32.1%)	6 (35.3%)	
Histological grade	1	2 (3.8%)	2 (11.8%)	0.344
	2	18 (34.0%)	7 (41.2%)	
	3	33 (62.3%)	8 (47.1%)	
Lymph node involvement	No	28 (52.8%)	10 (58.8%)	0.666
	Yes	25 (47.2%)	7 (41.2%)	
Distant metastasis	No	43 (81.1%)	13 (76.5%)	0.731
	Yes	10 (18.9%)	4 (23.5%)	
Lymphovascular invasion	Absent	31 (58.5%)	12 (70.6%)	0.372
	Present	22 (41.5%)	5 (29.4%)	
Perineural invasion	Absent	42 (79.2%)	15 (88.2%)	0.498
	Present	11 (20.8%)	2 (11.8%)	
Tumor necrosis	Absent	23 (43.4%)	10 (58.8%)	0.267
	Present	30 (56.6%)	7 (41.2%)	
Multifocality	Absent	44 (83.0%)	15 (88.2%)	0.728
	Present	9 (17.0%)	2 (11.8%)	

Continuous variables are presented as median (interquartile range, IQR). Categorical variables are presented as number (percentage). *p*-values are shown once per variable and correspond to comparisons between the LOW and HIGH HIF-1α expression groups.

**Table 4:** Association of clinicopathological features with distant metastasis.

Variable	Category	Metastasis Absent (n = 56)	Metastasis Present (n = 14)	*p* Value
OS death events, n (%)		2 (3.6%)	10 (71.4%)	<0.001
Median OS (months)		NR	84.0	<0.001
5-year OS (%)		96.4%	35.7%	<0.001
DFS events, n (%)		2 (3.6%)	14 (100%)	<0.001
Median DFS (months)		NR	18.0	<0.001
5-year DFS (%)		96.4%	0%	<0.001
Tumor size (cm)		2.5 (1.9–3.6)	3.1 (2.5–4.7)	0.264
Ki-67 (%)		50 (20–70)	60 (40–70)	0.575
SOD2 expression	Low	19 (33.9%)	3 (21.4%)	0.524
	High	37 (66.1%)	11 (78.6%)	
HIF-1α expression	Low	43 (76.8%)	10 (71.4%)	0.732
	High	13 (23.2%)	4 (28.6%)	
Combined phenotype	Both low	19 (33.9%)	3 (21.4%)	0.664
	Single high	24 (42.9%)	7 (50.0%)	
	Both high	13 (23.2%)	4 (28.6%)	
Stage group	Early (I–II)	47 (83.9%)	0 (0.0%)	<0.001
	Advanced (III–IV)	9 (16.1%)	14 (100.0%)	
Histological grade	1	3 (5.4%)	1 (7.1%)	0.815
	2	21 (37.5%)	4 (28.6%)	
	3	32 (57.1%)	9 (64.3%)	
Lymph node involvement	No	35 (62.5%)	3 (21.4%)	0.006
	Yes	21 (37.5%)	11 (78.6%)	
Lymphovascular invasion	No	38 (67.9%)	5 (35.7%)	0.027
	Yes	18 (32.1%)	9 (64.3%)	
Perineural invasion	No	47 (83.9%)	10 (71.4%)	0.277
	Yes	9 (16.1%)	4 (28.6%)	
Tumor necrosis	No	28 (50.0%)	5 (35.7%)	0.338
	Yes	28 (50.0%)	9 (64.3%)	
Multifocality	No	48 (85.7%)	11 (78.6%)	0.681
	Yes	8 (14.3%)	3 (21.4%)	
Molecular phenotype	CK5/6-positive TNBC	31 (55.4%)	7 (50.0%)	0.719
	CK5/6-negative TNBC	25 (44.6%)	7 (50.0%)	

NR = not reached (Not reached; median survival could not be calculated because more than half of the patients did not experience the event during follow-up).

**Table 5:** Cox proportional hazards regression for overall survival.

Covariate	HR (95% CI)	*p*
SOD2 high (vs. low)	3.78 (0.82–17.41)	0.088
HIF-1α high (vs. low)	2.85 (0.84–9.65)	0.092
Tumor size (per 1 cm)	1.17 (0.90–1.53)	0.241
Ki-67 (per 1%)	1.01 (0.98–1.03)	0.702
Grade 3 (vs. grade 1–2)	2.06 (0.56–7.62)	0.279
Node positive (vs. negative)	2.88 (0.77–10.71)	0.114
LVI present (vs. absent)	2.28 (0.68–7.66)	0.183
Distant metastasis (yes vs. no)	38.35 (4.87–302.25)	0.001
Advanced stage (III–IV vs. I–II)	9.45 (2.05–43.52)	0.004

Tumor size and Ki-67 were analyzed as continuous variables. Hazard ratios for tumor size represent the effect associated with each 1 cm increase, and hazard ratios for Ki-67 represent the effect associated with each 1% increase. Therefore, no discrete reference category applies to these variables.

**Table 6:** Multivariable Cox regression model for overall survival (Model A).

Covariate	HR (95% CI)	*p*
SOD2 high (vs. low)	1.90 (0.37–9.82)	0.443
HIF-1α high (vs. low)	2.40 (0.65–8.94)	0.191
Advanced stage (III–IV vs. I–II)	8.78 (1.85–41.78)	0.006

Tumor size and Ki-67 were analyzed as continuous variables. Hazard ratios for tumor size represent the effect associated with each 1-cm increase, and hazard ratios for Ki-67 represent the effect associated with each 1% increase. Therefore, no discrete reference category applies to these variables.

**Table 7:** Multivariable Cox regression model for overall survival (Model B).

Covariate	HR (95% CI)	*p*
SOD2 high (vs. low)	6.08 (0.67–55.24)	0.109
HIF-1α high (vs. low)	2.14 (0.26–17.74)	0.480
Tumor size (per 1 cm)	1.26 (0.82–1.94)	0.290
Grade 3 (vs. grade 1–2)	4.92 (0.51–47.53)	0.169
Node positive (vs. negative)	1.23 (0.07–22.02)	0.886
LVI present (vs. absent)	1.59 (0.34–7.34)	0.554
Distant metastasis (yes vs. no)	80.32 (4.97–1298.82)	0.002

Tumor size and Ki-67 were analyzed as continuous variables. Hazard ratios for tumor size represent the effect associated with each 1-cm increase, and hazard ratios for Ki-67 represent the effect associated with each 1% increase. Therefore, no discrete reference category applies to these variables.

**Table 8:** Cox proportional hazards regression for disease-free survival.

Covariate	HR (95% CI)	*p*
SOD2 high (vs. low)	2.63 (0.75–9.29)	0.133
HIF-1α high (vs. low)	1.71 (0.59–4.97)	0.324
Tumor size (per 1 cm)	1.13 (0.89–1.43)	0.314
Ki-67 (per 1%)	1.01 (0.99–1.03)	0.496
Grade 3 (vs. grade 1–2)	1.49 (0.52–4.30)	0.459
Node positive (vs. negative)	2.72 (0.94–7.84)	0.064
LVI present (vs. absent)	2.34 (0.85–6.45)	0.100
Advanced stage (III–IV vs. I–II)	18.76 (4.25–82.82)	<0.001

Tumor size and Ki-67 were analyzed as continuous variables. Hazard ratios for tumor size represent the effect associated with each 1-cm increase, and hazard ratios for Ki-67 represent the effect associated with each 1% increase. Therefore, no discrete reference category applies to these variables.

**Table 9:** Multivariable Cox regression model for disease-free survival (Model A).

Covariate	HR (95% CI)	*p*
SOD2 high (vs. low)	1.54 (0.39–6.02)	0.535
HIF-1α high (vs. low)	1.35 (0.43–4.23)	0.611
Advanced stage (III–IV vs. I–II)	17.49 (3.92–77.96)	<0.001

Tumor size and Ki-67 were analyzed as continuous variables. Hazard ratios for tumor size represent the effect associated with each 1-cm increase, and hazard ratios for Ki-67 represent the effect associated with each 1% increase. Therefore, no discrete reference category applies to these variables.

**Table 10:** Multivariable Cox regression model for disease-free survival (Model B).

Covariate	HR (95% CI)	*p*
SOD2 high (vs. low)	1.37 (0.29–6.46)	0.692
HIF-1α high (vs. low)	1.10 (0.20–6.16)	0.914
Tumor size (per 1 cm)	1.09 (0.79–1.51)	0.590
Grade 3 (vs. grade 1–2)	2.43 (0.52–11.23)	0.256
Node positive (vs. negative)	0.60 (0.10–3.68)	0.581
LVI present (vs. absent)	0.30 (0.07–1.23)	0.094
Distant metastasis (yes vs. no)	264.35 (23.65–2954.27)	<0.001

Tumor size and Ki-67 were analyzed as continuous variables. Hazard ratios for tumor size represent the effect associated with each 1-cm increase, and hazard ratios for Ki-67 represent the effect associated with each 1% increase. Therefore, no discrete reference category applies to these variables.

## Data Availability

The data supporting the findings of this study are available from the corresponding author upon reasonable request. The data are not publicly available because they contain information that could compromise patient privacy.
